# Assessing the accuracy of a machine learning prediction for 2 different shoulder prostheses: an external validation study

**DOI:** 10.1016/j.jseint.2025.04.024

**Published:** 2025-05-21

**Authors:** Gianluca Caprili, Andrea G. Calamita, Michele Novi, Domenico A. Campanacci, Simone Nicoletti

**Affiliations:** a Prosthetic Orthopedics, San Pietro Igneo Hospital, Fucecchio, FI, Italy; b Department of Orthopaedic Oncology, Careggi University Hospital, Firenze, Italy

**Keywords:** Reverse shoulder, Machine learning, Outcome prediction, Artificial intelligence, External validation, Glenohumeral osteoarthritis

## Abstract

**Background:**

The integration of machine learning in orthopedic surgery, including shoulder procedures, has garnered increasing interest. This retrospective analysis aims to externally validate a predictive analytics platform within a patient cohort undergoing reverse total shoulder arthroplasty for eccentric or concentric glenohumeral osteoarthritis.

**Methods:**

Ninety patients who underwent reverse total shoulder arthroplasty at our institution in 2022 were selected for this study. Patients were divided into 2 groups based on the type of implant (50 Exactech Equinoxe and 40 Zimmer Biomet Comprehensive). Preoperative evaluations included 19 variables per the tool requirements (demographics, diagnosis, comorbidities, patient-reported pain and function, and range of motion). The study compared the tool's outcome predictions with postoperative outcomes at 3-6 months, 1 year, and 2 years postsurgery for visual analog scale and active range of movement. We also quantified the mean absolute error (MAE) separately in the 2 groups and compared it to the MAE from the internal validation of the same tool.

**Results:**

Significant improvements in visual analog scale, active forward elevation, active abduction, and active external rotation met the minimal clinically important difference at 3-6 months, 1 year, and 2 years in both implant groups. Additionally, a modest improvement in active internal rotation was observed in both cohorts. In terms of MAE, we found a higher error than the internal validation only for forward elevation at 3-6 months in group 2 and a lower error in all the other outcome measures at all time points for both groups.

**Conclusion:**

The predictive analytics platform demonstrated a lower or similar MAE than the internal validation for both groups. Notably, we found that the tool's predictions are generalizable to another shoulder prosthesis, even though it was not trained on that particular product. This tool holds promise for aiding clinicians in managing patient expectations.

Interest in possible artificial intelligence applications in orthopedic surgery grew exponentially in the last 4 years.^13^^,^^16^ In particular, machine learning, given the large amount of data available in the previous decade, has been used in its many declinations (the most widely known is deep learning) to help clinicians in diagnostics and outcome predictions.^7^^,^^16^ In shoulder surgery, in particular, between 2010 and 2022, 45 studies regarding artificial intelligence applications in surgical and nonsurgical shoulder pathology were published,^5^ and the main research objects, as well as those where the best results were obtained, were imaging in diagnostics of rotator cuff pathology and prediction of functional outcomes in prosthetic surgery.^6^^,^^15^ We have therefore decided to externally validate, at our institution, a predictive analytics platform (Predict+ Rev B 12-0001480; Exactech, Gainesville, FL, USA) by comparing its predictions with our results at 3-6 months, 1 year, and 2 years postsurgery.

Predict+, an Exactech product, is defined as a “data-driven, clinical decision support tool that creates personalized patient outcome predictions using preoperative data to anticipate patients' postoperative results after anatomic or reverse total shoulder arthroplasty (rTSA)”.

This machine learning–powered predictive analytics platform was clinically released in 2020. Predict+ had been trained with a database of over 6,500 patients, two-third of which were used to create the prediction model and one-third to test the model^11^^,^^12^; of the 291 inputs recorded for each patient, the 19 that most influenced postoperative outcomes were selected. Visual analog scale (VAS), a range of motion, Global Shoulder Function, and Shoulder Arthroplasty Smart Score are predicted from 3 months to 7 years postoperative (Figs. 1 and 2).^11^^,^^12^

**Figure 1:**
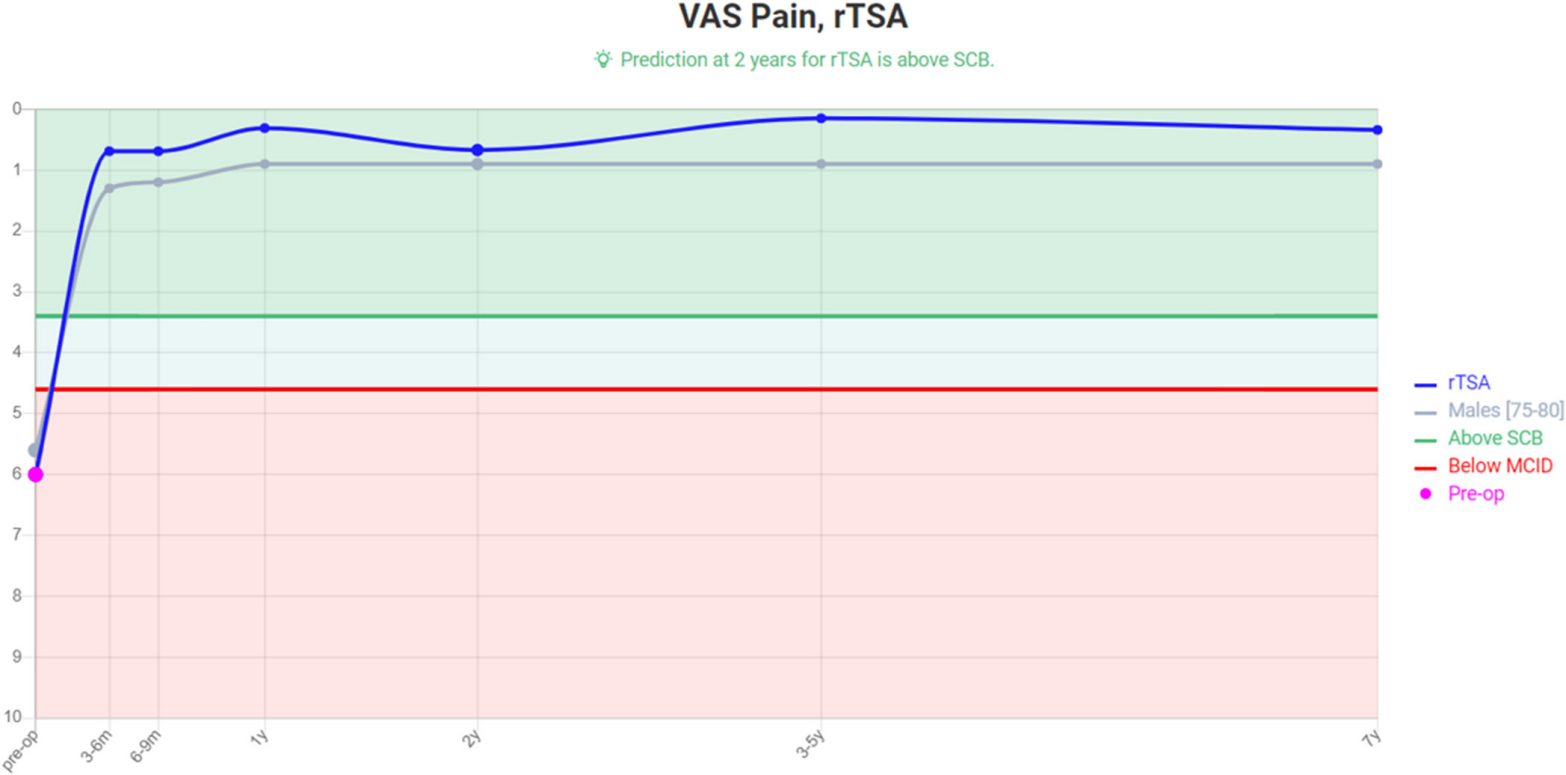
VAS values predictions from Predict+. *VAS*, visual analog scale; *MCID*, minimal clinically important difference; *rTSA*, reverse total shoulder arthroplasty; *SCB*, substantial clinical benefit.

**Figure 2:**
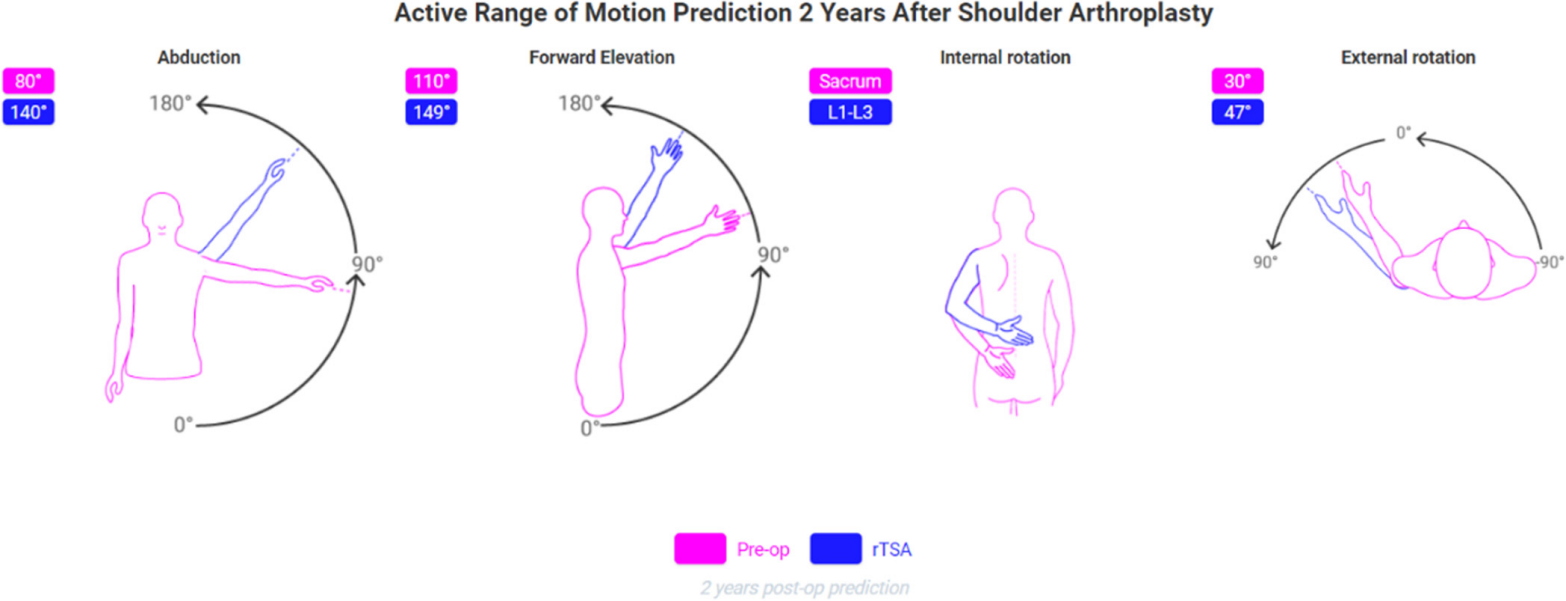
aROM predictions from Predict+. *aROM*, active range of motion.

## Materials and methods

All patients who underwent rTSA for glenohumeral osteoarthritis or cuff tear arthropathy in our institution in 2022 were eligible for inclusion in this study. Exclusion criteria were the absence of the preoperative inputs required by Predict+ (demographic data, diagnosis, comorbidities, patient-reported pain and function, ranges of motion), the absence of follow-up visits at 3-6 months, 1 year, and 2 years, and visits that do not include pain and range of motion measurements (Table I).

**Table I:** Inputs required by Predict+.

General information	Diagnosis, comorbidities
Patient information	Age, weight, height, gender, previous surgical operations on the shoulder, dominance
Range of motion and shoulder function	Active ROM (abduction, forward elevation, functional internal rotation, external rotation), passive external rotation, Global Shoulder Function score
Pain evaluation	VAS, pain at worst, pain when lying on the affected side, pain when touching the back of the neck, and pain when pushing with the affected arm

*rTSA*, reverse total shoulder arthroplasty; *ROM*, range of motion; *VAS*, visual analog scale.

A total of 90 patients met the inclusion criteria. The composition of the groups group 1, treated with Equinoxe rTSA (Exactech); group 2, treated with Comprehensive rTSA, (Zimmer Biomet, Warsaw IN, USA) was the following (Table II).

**Table II:** Group composition.

Subject	Group 1	Group 2
Number	50	40
Gender	26 F. 24 M	34 F. 6 M
Mean age	74.6	76.5

*F*, female; *M*, male.

The inputs above were inserted in Predict+, creating an anonymized file for every patient. Immediately after the insertion and confirmation of the inserted data, a prediction of the following clinical items at 6 time points after surgery (3 months, 6 months, 1 year, 2 years, 3-5 years, 7 years) is created by the tool: VAS score, Global shoulder function, Shoulder Arthroplasty Smart score, active abduction (aAB), active forward elevation (aFE), active external rotation (aER), and active functional internal rotation (aFIR).

Due to the retrospective nature of the study, we only considered the items of which we had a complete preoperative and postoperative evaluation at 3-6 months, 1 year, and 2 years, namely VAS, aAB, active forward flexion, aER, and aFIR.

The first data we evaluated were if the difference between pre and postoperative mean values in the 2 groups reached the minimal clinically important difference (MCID), namely: 1.4 points for VAS, 12° for aFE, 7° for aAB, and 3° for aER.^21^^,^^22^

We have not found an MCID value for aFIR^21^^,^^22^ in the current literature. We have therefore compared the mean values of our outcomes (at 3-6 months, 1 year, and 2 years postoperative, separately in the 2 groups) with Predict+ predicted outcomes, evaluating if the difference was below the MCID for VAS, active anterior flexion, aAB, and aER and measuring the difference between postoperative and predicted aFIR (aFIR values: 1 = lateral thigh; 2 = buttocks;3 = sacrum;4 = L4-L5; 5 = L1-L2; 6 = D10-D12; 7 = Higher than D10).

We also assessed the mean absolute error (MAE), namely the mean difference between the predicted and postoperative value for every outcome measure listed in Table III, separately in the 2 groups, at 3 different postoperative time points. MAEs we found were compared to the MAEs from the internal validation.^10^^,^^12^

**Table III:** Preoperative mean values in the 2 groups.

Preoperative values	Equinoxe (group 1)	Comprehensive (group 2)
VAS	6.1	6.4
Forward elevation	91°	92°
Abduction	75°	78°
External rotation	17°	17°
Functional internal rotation	2.8	2.7

*VAS*, visual analog scale.

## Results

Table III contains the preoperative mean values in the 2 groups:

There is no clinically significant difference between the 2 groups.

Table IV is the 3-6 months outcomes vs. Predict+ prediction at 3 months (average values):

**Table IV:** Three to 6 months outcomes vs. Predict+ prediction at 3 months (mean values).

3-6 mo outcomes	Group 1	Group 2
VAS	1.36 vs. 1 (PP)	1.05 vs. 0.9 (PP)
Forward elevation	129° vs. 119° (PP)	135° vs. 118° (PP)
Abduction	112° vs. 100° (PP)	110° vs. 101° (PP)
External rotation	26° vs. 27° (PP)	29° vs. 28° (PP)
Functional internal rotation	3.1 vs. 3.6 (PP)	2.9 vs. 3.4 (PP)

*VAS*, visual analog scale; *PP*, Predict+ predicted outcome.

As displayed in VAS (groups 1 and 2), forward elevation (group 1), and external rotation (groups 1 and 2), the difference between postoperative outcomes and Predict+ projections is below the MCID for those values.

Table V is the 1-year outcomes vs. Predict+ prediction at 1 year (average values):

**Table V:** One-year outcomes vs. Predict+ prediction at 1 year (mean values).

1-yr outcomes	Group 1	Group 2
VAS	0.8 vs. 0.7 (PP)	0.7 vs. 0.7 (PP)
Forward elevation	137° vs. 133° (PP)	144° vs. 135° (PP)
Abduction	122° vs. 109° (PP)	119° vs. 114° (PP)
External rotation	37° vs. 35° (PP)	30° vs. 37° (PP)
Functional internal rotation	3.8 vs. 4.1 (PP)	3.7 vs. 4 (PP)

*VAS*, visual analog scale; *PP*, Predict+ predicted outcome.

As shown, Predict+ projections deviated minimally (less than MCID) from postoperative outcomes in VAS (groups 1 and 2), anterior flexion (groups 1 and 2), abduction (group 2), and external rotation (group 1).

Table VI is the 2-year outcomes vs. Predict+ prediction at 2 years (average values):

**Table VI:** Two-year outcomes vs. Predict+ prediction at 2 years (mean values).

2-yr outcomes	Group 1	Group 2
VAS	0.8 vs. 0.7 (PP)	0.7 vs. 0.7 (PP)
Forward elevation	139° vs. 135° (PP)	145° vs. 137° (PP)
Abduction	123° vs. 109° (PP)	119° vs. 115° (PP)
External rotation	39° vs. 37° (PP)	32° vs. 37° (PP)
Functional internal rotation	4.0 vs. 4.2 (PP)	3.8 vs. 4.1 (PP)

*VAS*, visual analog scale; *PP*, Predict+ predicted outcome.

As shown, deviations below the MCID were found in VAS (groups 1 and 2), anterior flexion (groups 1 and 2), abduction (group 2), and external rotation (group 1).

Table VII is the MAE in the 2 groups at all time points for every outcome measure, separately compared with the MAE from the internal validation:

**Table VII:** MAE in the 2 groups at all time points for every outcome measure, separately compared with the MAE from the internal validation.

Subject	MAE (group 1)	MAE (group 2)	MAE (internal validation)	% Of the difference between group 1 and internal validation	% Of the difference between group 2 and internal validation
VAS (3-6 mo)	1.16	0.95	1.6	27.5% better	40.6% better
VAS (1 yr)	0.70	0.625	1.6	56.2% better	60.9% better
VAS (2 yr)	0.75	0.80	1.5	50% better	46.7% better
Forward elevation (3-6 mo)	20.4	23.75	21.9	6.8% better	8.4% worse
Forward elevation (1 yr)	16.2	14.5	18.7	13.3% better	22.5% better
Forward elevation (2 yr)	16.6	15.2	18.9	12.2% better	19.6% better
Abduction (3-6 mo)	21.6	22.25	22.5	4% better	1% better
Abduction (1 yr)	20.6	14.25	23.3	11.6% better	38.8% better
Abduction (2 yr)	19.6	18.6	19.7	0.5% better	5.6% better
External rotation (3-6 mo)	12.7	10.25	12.8	0.8% better	19.9% better
External rotation (1 yr)	7.3	8.6	12.4	41.1% better	30.5% better
External rotation (2 yr)	7.1	8.1	12.1	41.3% better	33.1% better
Internal rotation (3-6 mo)	1.02	0.63	1.09	6.4% better	42.6% better
Internal rotation (1 yr)	0.62	0.6	1.28	51.6% better	53.1% better
Internal rotation (2 yr)	0.71	0.65	1.18	39.8% better	44.9% better

*VAS*, visual analog scale; *MAE*, mean absolute error.

To summarize, in group 1 (Equinoxe), Predict+ projected outcomes diverged less than MCID from actual outcomes in VAS at 3-6 months, at 1 year, and 2 years; anterior flexion at 3-6 months, at 1 year, and 2 years, and external rotation at 3-6 months, at 1 year, and 2 years; in group 2 (Comprehensive) similar accuracy was found in VAS at 3-6 months, at 1 year, and 2 years; anterior flexion at 1 year and 2 years, abduction at 1 year and 2 years, and external rotation at 3-6 months. In terms of MAE, we found good results in both groups, similar to or better than those of internal validation.

## Discussion

The primary endpoint of our study was to externally validate a specific prediction tool in rTSA. We calculated the difference between expected and postoperative average values of the 2 groups and the MAE considering every patient at every time point. Regarding the difference between mean values, we found the best accuracy in VAS and active forward flexion prediction, as well as for internal rotation, despite the lack of a recognized MCID; our results are consistent with previous literature.^4^^,^^9^^,^^11^^,^^17^^,^^18^

The MAE results for both groups are certainly encouraging, as they are lower than those of the internal validation. We found a MAE similar to, lower than, or markedly lower (up to 60.9% for VAS at 1 year in group 2) compared to that of the internal validation study. Simmons et al, in an external validation of the same tool (only for the Equinoxe implant, group 1), found similar good results regarding MAE.^19^

Another study^20^ assessed the possibility that a surgeon might change the surgical indication (rTSA vs. anatomic total shoulder arthroplasty) after consulting a Clinical Decision Support Tool: we did not evaluate this aspect, but we believe that this tool may be more patient-oriented than surgeon-oriented, precisely because of the prevalence of pessimistic rather than optimistic predictions.

We also compared the accuracy of predictions for Equinoxe implants, for which the tool was developed, with the accuracy of predictions for a different implant by another company. We did not find significant differences in the accuracy of predictions, possibly because the 19 input parameters used affect outputs the same way with a different implant.^12^ The observation of a MAE in group 2 (Zimmer Biomet Comprehensive), similar to that of group 1 (Exactech Equinoxe), and comparable to or lower than previous internal and external validations is, in our opinion, a significant result.^10^^,^^12^^,^^19^ This is the first study where the Predict+ tool has been used for an implant for which it was not trained, and we can say that the tool gave similar results in predicting functional outcomes even when used for an implant from a different company and with different characteristics: Exactech Equinoxe is classified as a lateralized humerus/medialized glenoid implant, while Zimmer Biomet Comprehensive is classified as a lateralized humerus/lateralized glenoid implant.^23^ In addition, we didn't find significant differences in postoperative results between the 2 groups.

Preoperative ROM is the primary determinant of postoperative ROM, particularly for active anterior flexion.^1^, ^2^, ^3^^,^^8^ Postoperative external and internal rotation is also determined by factors not considered by the tool, such as teres minor atrophy (for external rotation)^3^ or greater tuberosity distalization (for functional internal rotation)^8^ his could explain why we found better results in aFE predictions.

In the near future, predictions based on preoperative parameters may be integrated with intraoperative data collected by computer-assisted surgery tools and with apps able to quantify a patient's ROM reproducibly and with tools that classify preoperative and postoperative radiographs (already validated).^24^ The better quality of the data provided will improve the training phase of machine learning tools and their accuracy. However, a recent study on complications after rTSA stated that patient-specific risk factors (those counted by our tool) are more strictly associated with complications rate than implant design and surgical technique.^14^

The main strength of our study is the comparison, for the first time, between the predictions for 2 different prosthetic implants, which furthermore gave similar and reliable results even though the tool was not trained on the implant used in group 2.

As for the limitations, our analysis was carried out on a limited patient cohort, and due to its retrospective nature, we could only study the outcomes that were constantly present in our outpatient visits (VAS and active range of motion). Moreover, due to the short duration of our follow-up (no more than 2 years), we could not fully utilize Predict+, which provides predictions for up to 7 years. Another limitation is that the sample size of the 2 groups was different (50 vs. 40), and there were differences in the timing of the first postoperative visit. However, all patients had 1 visit within the 3-6 month range.

## Conclusion

In our experience, Predict+ provided similar or better results compared to previous literature for VAS and active range of motion predictions after rTSA, even for an implant which was not used during the training phase of the outcome prediction tool. In our opinion, the tool can be advantageous in managing patient expectations and enhancing adherence to rehabilitation protocols. Further investigations are needed to externally validate this tool, and its accuracy can be further increased with the aid of other machine learning–based programs.

## Disclaimers:

Funding: No funding was disclosed by the authors.

Conflicts of interest: The authors, their immediate families, and any research foundations with which they are affiliated have not received any financial payments or other benefits from any commercial entity related to the subject of this article.
